# Does the type of pharmacy used influence medication adherence? A retrospective observational study in Aotearoa, New Zealand

**DOI:** 10.1177/17151635241267025

**Published:** 2024-07-28

**Authors:** James Nind, Carlo A. Marra, Shane Scahill, Charlotte Siermans, Alesha Smith

**Affiliations:** School of Pharmacy, University of Otago, Dunedin, New Zealand; School of Pharmacy, University of Otago, Dunedin, New Zealand; School of Pharmacy, University of Auckland, Auckland, New Zealand; School of Pharmacy, University of Otago, Dunedin, New Zealand; School of Pharmacy, University of Otago, Dunedin, New Zealand

## Abstract

**Background:**

Community pharmacies in New Zealand have varying ownership and operational structures. Unlike independent pharmacies, corporate and hybrid pharmacies do not charge prescription copayments.

**Objectives:**

This research aimed to determine whether people receiving free prescriptions from corporate and hybrid pharmacies (via copayment waiver) have greater medication adherence than the users of independent pharmacies.

**Methods:**

A nationwide, retrospective, observational study linked 1 year of dispensing data (1/05/2022 to 30/04/2023) from the Pharmaceutical Collection to patient enrollment data using a National Health Index number to identify demographics of different pharmacy-type users. People were assigned to a particular type of pharmacy if they collected at least 70% of their prescriptions from there; if they did not meet this threshold, they were defined as mixed users. People were classified as adherent if dispensing data showed they collected their supply of medication to cover at least 80% of the study period.

**Results:**

The sample captured 218,080 people taking at least 1 diabetes medication, with a total of 360,079 unique medications being included in the analysis. The majority, 156,893, used independent pharmacies. The type of pharmacy used was shown to be a significant predictor of adherence. Corporate and hybrid pharmacy users were 0.90 (95% CI 0.88 to 0.93) and 0.93 (95% CI 0.90 to 0.96) times as likely be adherent than the users of independent pharmacies. Mail order pharmacy users were the most likely to be adherent, whereas mixed pharmacy users were the least likely to be adherent.

**Conclusions:**

Our findings suggest that prescription copayments provided by corporate and hybrid pharmacies are not the most significant barrier to medication adherence. Further research may identify more efficient ways of improving medication adherence than removing prescription copayments for all.

**Knowledge into Practice**

- A variety of models of community pharmacy are found in New Zealand, several of which have used price competition strategies and have not been charging prescription copayments in order to offer free prescriptions.
- Although prescription copayments have been identified as a barrier to medication adherence, it is not known whether patients using pharmacies that do not charge prescription copayments have better medication adherence.
- Significant differences in the rates of medication adherence were observed between the users of different types of pharmacy.
- The findings of this study suggest that differences in how pharmacies operate may have a greater influence on medication adherence than prescription copayments.

## Introduction

Community pharmacists are among the most frequently seen health professionals; it is estimated that patients see pharmacists up to 10 times more frequently than general practitioners.^1^ When combined with their expertise in medicines, pharmacists are positioned to positively influence their patients’ medication adherence.^2,3^

Medication adherence describes the extent to which a patient’s behaviour follows the agreed-upon recommendations from a health care provider and commonly includes taking a medication as prescribed.^4^ Poor medication adherence is associated with poor health outcomes for patients and high costs to the health system due to inefficiencies.^4,5^ Type 2 diabetes mellitus (T2DM) was chosen as the focus of this study because it is a chronic lifelong condition with serious consequences if poorly controlled.^6,7^ In addition, T2DM has been the focus of several international studies comparing the effects of pharmacy types on patients’ adherence.^8-10^

**Figure 1:**
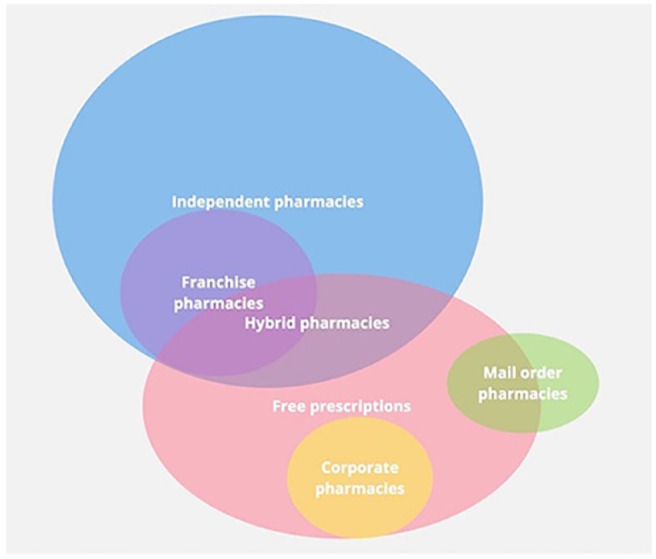
Venn diagram showing different types of pharmacies

This study discusses several types of community pharmacy: independent, corporate, hybrid and mail order, which are defined in Table 1.^11,12^ Figure 1 was created to display the overlap in characteristics such as ownership and prescription copayments between the pharmacy types.

**Table 1:** Pharmacy type definitions

Pharmacy type	Definition	Copayment status
Independent	A pharmacy that is owned by an individual proprietor or small group.A subset of these, often called *franchise pharmacies*, pay for the support and recognition of a brand such as Unichem or Life, and have not been considered in this study’s analysis.	No free prescriptions.
Corporate	A pharmacy part of a chain owned by a corporation such as Chemist Warehouse, Countdown or Bargain Chemist.	Offers free prescriptions.
Hybrid	A term coined for this study to describe an independently owned pharmacy that offers free prescriptions.	Offers free prescription.
Mail order	A type of pharmacy that delivers the majority of its prescriptions to its patients.	Within New Zealand, some offer free prescriptions, delivery and packing for individuals taking ≥4 regular medications.^11,12^

Key differences in the consumer profiles of each type of pharmacy were reported in a recent study.^13^ Corporate pharmacies were granted licences to operate, in part, on the premise that they would improve access to medicines for Māori, the Indigenous people of New Zealand, and Pacific peoples; there is no evidence that these groups prefer these pharmacies.^13,14^ Māori made up relatively equal proportions of users across all pharmacy types, whereas hybrid pharmacies served a greater proportion of Pacific peoples and those from areas of higher deprivation than other pharmacy types.^13^

**Mise En Pratique Des Connaissances**

- Il existe différents modèles de pharmacies communautaires en Nouvelle-Zélande; certains d’entre eux ont eu recours à des stratégies de concurrence des prix et n’ont pas facturé de quote-part afin de délivrer gratuitement les médicaments sur ordonnance.
- Bien que les quotes-parts d’ordonnance aient été désignées comme étant un obstacle à l’observance thérapeutique, nous ne savons pas si les clients de ces pharmacies ne facturant pas de quotes-parts d’ordonnance ont une meilleure observance des médicaments.
- Des différences significatives dans les taux d’observance de la prise de médicaments ont été observées entre les utilisateurs des différents types de pharmacies.
- Les résultats de cette étude suggèrent que les différences dans la façon dont les pharmacies fonctionnent peuvent avoir une plus grande influence sur l’observance de la prise des médicaments que l’éventuelle application des quotes-parts d’ordonnance.

A recent systematic review examined the international literature comparing medication adherence between the users of chain pharmacies—a similar model to New Zealand’s corporate pharmacies—and independent pharmacies; the authors of that review were unable to substantiate a difference in medication adherence between the type of pharmacy used.^15^

Prescription copayments vary between the different pharmacy types in New Zealand. During the study period, patients paid $5 NZD ($4.16 CAD) as a copayment for most prescriptions, or $15 NZD ($12.48 CAD) for specialist and dental prescriptions, for their first 20 prescription items in the calendar year.^16,17^

From July 1, 2023, standard $5 NZD prescription copayments were removed as part of New Zealand’s plans to reduce barriers to accessing medicine.^18^ By comparing those individuals already receiving free prescriptions via corporate and hybrid pharmacies to those paying for prescriptions at independent pharmacies, we may anticipate the potential impact of removing prescription copayments on equitable access to medication.

This research primarily aims to compare the rates of adherence to diabetes medications between the users of New Zealand’s different types of pharmacies. This will provide insight into the effect that removing prescription copayments may have on people’s medication adherence. We hypothesized that people receiving free prescriptions would have greater medication adherence than those who pay prescription copayments.

## Methods

We followed the Strengthening the Reporting of Observational Studies in Epidemiology (STROBE) cohort reporting guideline when designing this study.

### Ethics

This study received ethical approval from the University of Otago ethics committee (HD23/056). Individual patient consent is not required to use these data, because anonymity is preserved through the use of the encrypted National Health Index (NHI).

### Data source/overview

Study data were collected from the Pharmaceutical Collection, which captures all of New Zealand’s community-dispensed medicines except those given as a hospital inpatient. This was linked to Primary Health Organisation (PHO) information on patient demographics via encrypted NHI and to pharmacy characteristic information that the research team extracted from Healthpoint. This is a national health services directory designed to provide people with up-to-date information about how to access health services, from the pharmacy’s name.^19,20^

### Participant selection

A retrospective, observational, cohort study design was carried out to capture everyone who collected a prescription for a diabetes medication between May 1, 2022, and April 30, 2023.

The following medications were studied: dulaglutide, empagliflozin with or without metformin, metformin, pioglitazone, sulfonylureas and vildagliptin with or without metformin. Insulin was excluded from analysis because it is dispensed in whole vials, potentially leading to an overestimation of people’s medication adherence. Liraglutide was excluded because it only became funded on March 1, 2023.

Demographic information regarding age, ethnicity, deprivation quintile and sex were extracted from the PHO dataset. Individuals were grouped into age bands by their age at the last date of dispensing in the study period. Ethnicity was self-reported, and people were grouped into 1 of the following categories: Māori, Pacific People, Asian and New Zealand European/other using prioritization in the order given above.^21^ For example, if someone identified as Māori and New Zealand European, they were recorded as Māori. Deprivation quintiles were based on individuals’ addresses using the New Zealand Index of Deprivation (NZDep).^22^ The levels were condensed into quintiles 1 through 5, quintile 1 being the least deprived area and 5 being an area with the most deprivation.^22^ Sex was self-reported as male, female, unknown or other. Medication burden was crudely measured by performing a count of the number of unique medications when an individual collected 2 or more original prescriptions during the study period; this was then grouped into bands of 0–4, 5–9 or more than 9, loosely following the definitions for polypharmacy and hyperpolypharmacy.^23^

### Community pharmacy selection

A list of New Zealand’s community pharmacies and their relevant characteristics was created using Healthpoint. Pharmacies were categorized into 1 of 4 groups: corporate, mail order, independent or hybrid. Pharmacies that were part of Chemist Warehouse, Bargain Chemist or Countdown brands were classified as corporate pharmacies. Zoom, PillDrop and Pharmacy Direct were grouped as mail order pharmacies. The remaining pharmacies were classified as hybrid if they advertised free prescriptions on Healthpoint or independent if not. Pharmacies were contacted regarding prescription copayments if the information on Healthpoint was ambiguous.

### Data analysis

Individuals were grouped based on the type of pharmacy from which they collected at least 70% of their prescriptions. If they did not meet this criterion for any type of pharmacy, they were assigned as a mixed pharmacy type user. Sensitivity testing was performed during a previous study to determine whether a greater percentage cut-off point (≥75%, ≥80%) should be applied, but the number of users of each type of pharmacy did not differ significantly.^24^ Pearson chi-square tests were used to test the significance of the differences between the demographics of each type of pharmacy user.

**Figure 2:**
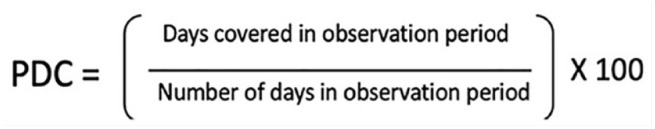
Formula for proportion of days covered

Medication adherence was calculated as the proportion of days covered (PDC), where the number of days’ supply of medicine a person received during the study period was divided by 365, the number of days in the study period, as shown in Figure 2.

The PDC was calculated for each medication and recorded as adherent if the PDC was 80% or higher, a threshold beyond which adherence is considered good.^4,25,26^ For example, an individual could be adherent to 2 of their 3 medications.

Because the probability of an individual being adherent to 1 medication is not independent of the probability of their being adherent to a separate medication studied, a mixed-effects logistic regression model was generated using the GLMer function in the statistical package RStudio.^27,28^ In this model, the type of pharmacy a person used and their age, sex, deprivation index, ethnicity and medication burden were tested as predictors of someone being adherent to a medication. To account for the multiple medications some people took, which were each treated as separate observations, individuals, identified by unique encrypted NHI, were treated as a random effect.

## Results

Between May 2022 and April 2023, a total of 218,080 people collected at least 1 prescription for a diabetes medication, resulting in people taking a combined 360,079 unique medications that were included in analysis.

Although independent pharmacies were used by the majority of people with diabetes (*n* = 156,893), a significant number of people were classified as corporate (*n* = 27,098), hybrid (*n* = 17,311) or mixed (*n* = 15,368) pharmacy users. Mail order pharmacy users made up a small proportion of our study (*n* = 1410).

Several differences were observed between the demographics of the users of each type of pharmacy. Hybrid pharmacies had a greater proportion of users living in the most deprived areas (quintile 5), 55.5%, and Pacific peoples, 36.1%, than other pharmacy types. Corporate pharmacies had a greater proportion of Asian users, 36.2%, than other types of pharmacies. A greater proportion of mixed pharmacy users were taking less than 5 medications during the study period, 45.6%, compared with other pharmacy users. Mail order pharmacy users had a greater medication burden than other pharmacy types, with 92.9% taking 5 or more medications regularly. Independent pharmacies had the greatest proportion of New Zealand European/other users, 59.3%, and had a similar profile of older users with a greater medication burden compared with mail order pharmacies, even though independent pharmacies had over 10 times more users. No meaningful differences were observed between sexes. The full demographic profile for each type of pharmacy user is displayed in Table 2.

**Table 2:** Demographic breakdown of the individuals using each type of pharmacy

	Corporate	Hybrid	Mail order	Mixed	Independent	Total	*p*-value*
Total	27,098 (12.4)^†^	17,311 (7.9)	1410 (0.6)	15,368 (7.0)	156,893 (71.9)	218,080	
Deprivation							
1 (least deprived)	4403 (16.2)	1125 (6.5)	189 (13.4)	1610 (10.5)	21,307 (13.6)	28,634	<0.001
2	4667 (17.2)	1532 (8.8)	231 (16.4)	2107 (13.7)	24,246 (15.5)	32,783	<0.001
3	4670 (17.2)	2020 (11.7)	297 (21.1)	2329 (15.2)	28,046 (17.9)	37,362	<0.001
4	6259 (23.1)	3023 (17.5)	294 (20.9)	3298 (21.5)	35,964 (22.9)	48,838	<0.001
5 (most deprived)	7099 (26.2)	9611 (55.5)	399 (28.3)	6024 (39.2)	47,330 (30.2)	70,463	<0.001
Age							
13–24 years	296 (1.1)	114 (0.7)	0 (0.0)	294 (1.9)	1099 (0.7)	1803	<0.001
25–40 years	3124 (11.5)	1530 (8.8)	45 (3.2)	2712 (17.7)	9386 (6.0)	16,797	<0.001
41–55 years	7283 (26.9)	4454 (25.7)	258 (18.3)	4174 (27.2)	29,843 (19.0)	46,012	<0.001
56–70 years	10,955 (40.4)	7479 (43.2)	577 (40.9)	5428 (35.3)	63,079 (40.2)	87,518	<0.001
71–85 years	5153 (19.0)	3467 (20.0)	487 (34.5)	2574 (16.7)	47,727 (30.4)	59,408	<0.001
>85 years	287 (1.1)	267 (1.5)	43 (3.0)	186 (1.2)	5759 (3.7)	6542	<0.001
Sex							
Female	13,526 (49.9)	8600 (49.7)	671 (47.6)	7906 (51.4)	73,522 (46.9)	104,225	<0.001
Male	13,551 (50.0)	8704 (50.3)	739 (52.4)	7449 (48.5)	83,262 (53.1)	113,705	<0.001
Other	5 (0.0)	2 (0.0)	0 (0.0)	0 (0.0)	12 (0.0)	19	0.507
Unknown	16 (0.0)	5 (0.0)	0 (0.0)	13 (0.0)	97 (0.0)	131	0.351
Ethnicity							
Asian	9813 (36.2)	5160 (29.8)	260 (18.4)	4568 (29.7)	18,931 (12.1)	38,732	<0.001
European/other	9776 (36.1)	3353 (56.5)	800 (56.7)	4739 (30.8)	93,044 (59.3)	111,712	<0.001
Māori	3744 (13.8)	2544 (14.7)	256 (18.2)	2690 (17.5)	27,370 (17.4)	36,604	<0.001
Pacific peoples	3765 (13.9)	6254 (36.1)	94 (6.7)	3371 (21.9)	17,548 (11.2)	31,032	<0.001
Medication burden^‡^							
0–4	10,188 (37.6)	4977 (28.7)	99 (7.0)	7009 (45.6)	45,404 (28.9)	67,677	<0.001
5–9	12,396 (45.7)	8125 (46.9)	707 (50.1)	6033 (39.3)	71,013 (45.3)	98,274	<0.001
≥10	4514 (16.7)	4209 (24.3)	604 (42.8)	2326 (15.1)	40,476 (25.8)	52,129	<0.001
Mean number of diabetes medications each user takes	1.65	1.81	1.78	1.60	1.64	1.65	

* The probability that differences in the proportion of the demographic using each type of pharmacy are the result of chance.

† Values in parentheses show the percentage of the demographic category which uses that type of pharmacy.

‡ Number of unique medications when a person collected ≥2 original prescriptions for any type of medication during the study period, which served as a proxy measure of medication burden.

The distribution of diabetes medications taken by the users of each type of pharmacy is displayed in Table 3. Although of statistical significance, the observed differences were not deemed to have a meaningful impact on the results.

**Table 3:** Distribution of diabetes medications taken by the user of each type of pharmacy

	Corporate	Hybrid	Mail order	Mixed	Independent	Total	*p*-value*
Total	44,671 (12.4)^†^	31,254 (8.7)	2516 (0.7)	24,638 (6.8)	257,000 (71.4)	360,079	
Dulaglutide	2174 (4.9)	977 (3.1)	174 (6.9)	1058 (4.3)	14,181 (5.5)	18,564	<0.001
Empagliflozin with/without metformin	7840 (17.6)	6481 (20.7)	501 (19.9)	4180 (16.9)	45,865 (17.8)	64,867	<0.001
Metformin	18,646 (41.7)	11,144 (35.7)	910 (36.2)	11,030 (44.8)	109,580 (42.6)	151,310	<0.001
Pioglitazone	282 (0.6)	209 (0.7)	21 (0.8)	141 (0.6)	1662 (0.6)	2315	<0.001
Sulfonylureas	5219 (11.7)	4652 (14.9)	339 (13.5)	2741 (11.1)	29,797 (11.6)	42,748	<0.001
Vildagliptin with/without metformin	10,510 (23.5)	7791 (24.9)	571 (22.7)	5488 (22.3)	55,915 (21.8)	80,275	<0.001

* The probability that differences in the proportion of the demographic using each type of pharmacy are the result of chance.

† Values in parentheses show the percentage of the demographic category which uses that type of pharmacy.

Using PDC as a measure of adherence, we found that overall, 68.2% of diabetes medications had a PDC 80% or higher. The rate of medication adherence was greatest from mail order pharmacy users, with a medication adherence rate of 82.6%. Independent and hybrid pharmacy users also had above-average medication adherence rates, 69.4% and 68.4%, respectively. Corporate pharmacy users had a below-average adherence rate of 64.9% and mixed pharmacy users had the lowest rate of medication adherence at 60.2%.

After we accounted for the demographic differences in each of the pharmacies’ users, the type of pharmacy someone used had a statistically significant influence on that person’s likelihood of being adherent to their medication. Corporate pharmacy users were 0.90 (95% CI 0.88 to 0.93) times as likely to be adherent than the users of independent pharmacies. Similarly, the users of hybrid pharmacies were 0.93 times (95% CI 0.90 to 0.96) as likely to be adherent. Mail order pharmacy users had the greatest rate of medication adherence; they were 1.59 (95% CI 1.42–1.79) times as likely to be adherent compared with an independent pharmacy user. Those we defined as mixed pharmacy users were the least likely to be adherent and were 0.81 (95% CI 0.79–0.84) times as likely to be adherent compared with an independent pharmacy user.

Several demographic variables were also shown to be predictors of medication adherence. Men were 1.09 (95% CI 1.08–1.11) times more likely to be adherent than women.

A positive relationship was noted between age and medication adherence. The younger age bands (defined as 13–24, 25–40 and 40–55 years old) were less likely to be adherent than the 55- to 70-year-old reference age band, but those aged 71–85 years were more likely to be adherent.

Asian and Pacific peoples were not significantly more or less likely to be adherent than New Zealand European/other users. However, Māori were 0.88 (95% CI 0.86–0.90) times as likely to be adherent as New Zealand European/other users.

Medication burden was the most significant predictor of adherence: the more unique medicines someone took, the more likely they were to be adherent. Individuals taking 5 to 9 medications were 2.91 (95% CI 2.84–2.97) times more likely to be adherent than those taking 0 to 4 medicines; similarly, those taking more than 9 medications were 4.30 (95% CI 4.19–4.40) times as likely.

The type of medicine was a significant predictor of adherence. People were significantly more likely to be adherent to empagliflozin, sulfonylureas, pioglitazone and vildagliptin than metformin. They were less likely to be adherent to dulaglutide than metformin.

The deprivation level of the area where someone lives was not found to be a significant predictor of adherence.

Table 4 displays the odds ratios, confidence intervals and significance of each demographic category with respect to being adherent to the given reference category.

**Table 4:** Mixed effects logistic regression of study variables in relation to medication adherence

Variable	Odds ratio	95% Confidence interval	*p*-value
Type of pharmacy used			
Independent	Reference	—	—
Corporate	0.90	0.88–0.93	<0.001
Hybrid	0.93	0.90–0.96	<0.001
Mail order	1.59	1.42–1.79	<0.001
Mixed	0.81	0.79–0.84	<0.001
Deprivation			
Quintile 1 (least deprived)	0.97	0.94–1.01	0.104
Quintile 2	0.99	0.96–1.02	0.606
Quintile 3	Reference	—	—
Quintile 4	1.00	0.97–1.03	0.921
Quintile 5 (most deprived)	0.98	0.96–1.01	0.141
Sex			
Female	Reference	—	—
Male	1.09	1.08–1.11	<0.001
Other	0.27	0.09–0.78	0.016
Sex unknown	0.81	0.59–1.12	0.206
Age			
13–24 years	0.31	0.28–0.34	<0.001
25–40 years	0.54	0.52–0.56	<0.001
41–55 years	0.92	0.90–0.94	<0.001
56–70 years	Reference	—	—
71–85 years	1.05	1.02–1.07	<0.001
>85 years	1.01	0.96–1.07	0.627
Ethnicity			
New Zealand European/other	Reference	—	—
Asian	1.01	0.98–1.03	0.542
Māori	0.88	0.86–0.90	<0.001
Pacific peoples	1.01	0.98–1.03	0.606
Medication burden			
0–5	Reference	—	—
5–9	2.91	2.84–2.97	<0.001
>9	4.30	4.19–4.40	<0.001
Medication			
Metformin	Reference	—	—
Dulaglutide	0.81	0.78–0.84	<0.001
Empagliflozin with/without metformin	1.27	1.24–1.30	<0.001
Pioglitazone	1.58	1.42–1.76	<0.001
Sulfonylureas	1.93	1.87–1.98	<0.001
Vildagliptin with/without metformin	1.44	1.41–1.47	<0.001

## Discussion

The results of this study do not support our hypothesis that users of corporate and hybrid pharmacies who waive prescription copayments have greater rates of medication adherence than the users of independent pharmacies. Instead, those receiving free prescriptions from these types of pharmacies may actually have lower rates of medication adherence than those who pay prescription copayments.

The finding that corporate and hybrid pharmacy users were less likely to be adherent suggests that prescription copayments may not be the greatest barrier to accessing medicine. Although there is a base of evidence, both internationally and in New Zealand, linking reduced prescription copayments with improved medication adherence, the effects may be less significant when copayments are very low.^3,5,29,30^

Several randomized controlled trials have investigated removing relatively low prescription copayments for medications such as statins and antihypertensives. Adherence improved by 3% to 6% when prescription copayments were removed; however, the rate of major cardiovascular events, the primary outcomes, did not decrease.^31-33^ A New Zealand study reported a reduction in hospitalizations by removing prescription copayments and hypothesized that this was due to improved patient adherence, although medication adherence was not measured.^34^

These findings add to the base of evidence that factors other than prescription copayments likely have a greater effect on the rates of medication adherence, questioning the effectiveness of the decision to remove prescription copayments for all. Similarly, the finding that deprivation was not a predictor of adherence indicates either that the measures of deprivation are ineffective at identifying groups who need additional support or that deprivation is not a significant barrier. As such, other strategies may be more efficient at targeting interventions aimed at improving medication adherence and access.

It has been suggested in both international literature and New Zealand’s trade publications that the quality of care differs between independent and corporate-owned pharmacies, which may have a greater positive influence on medication adherence.^35-38^ Should prescriptions remain free, repeating this study would allow for a more direct comparison between the service offered by each type of pharmacy without inference from the price of prescriptions. However, there may be innate personal characteristics that attract people who are less likely to be adherent to their medication to pharmacies that offer free prescriptions that pharmacy records data, such as that used in this study, are unable to measure.

The positive effect that increased medication burden had on adherence was an interesting finding not generally supported in the literature.^3,8,39^ A potential explanation for this finding would be the association with spending more time with health professionals, which is understood to have a positive influence on adherence.^3,8^

Similarly, low rates of medication adherence to diabetes medications have been reported in New Zealand. Horsburgh et al.^7^ reported that just 63% of study members were adherent to metformin, and a 2015 meta-analysis of 12 studies reported 67.9% of patients to be adherent.^40^

Although poor medication adherence and access to medication are not novel to New Zealand or diabetes, caution must be taken when generalizing the findings of this study to both the general population and other health systems.^41,42^ Similar rates of adherence have been reported between the users of independent and chain pharmacies, which have a comparable operating model to New Zealand’s corporate pharmacies, according to Canadian and American studies on statins, diabetes and chronic disease medications.^8,43^

Further research into how people choose pharmacies may help to identify a more effective way of improving medication adherence and access to medicine than simply removing prescription copayments, which the current study suggests may not make a significant impact to improve medication adherence and access to long-term medicines, such as those used in diabetes.

### Strengths and limitations

A strength of this study is that it included the entire population of people who had collected a diabetes medication during the study period, thus avoiding sampling bias. The method used is not subjective to recall bias or socially desirable responding, which can be an issue with self-reported measures of adherence.

There are several limitations to our study design. First, the method could not determine whether people started, stopped or switched medications following advice from their physician during the observation period. If they did so, they were less likely to be recorded as adherent because the number of days’ supply they received was still divided by the 365-day supply period. This likely would result in medication adherence rates being underestimated; however, there is no evidence to suggest that the rates of starting, stopping or switching medications differ between users of different types of pharmacies. Additionally, although people are likely to have a supply of medication prior to the start of the study period, it balances against any medication they have remaining at the end of the year and will occur equally across all types of pharmacies.

Second, the information taken from Healthpoint to classify pharmacies was taken at a single point in time and may not have been up-to-date, as some pharmacies may have only operated as a hybrid pharmacy for some of the study period, potentially reducing the accuracy of our results. The arbitrary 80% threshold used to classify medication adherence is also a limitation; different medications may have different requirements to be effective. A common assumption in using pharmacy records to estimate medication adherence is that a medication collected is a medication taken.^44^ However, people may be less likely to take a medication correctly if they did not pay for it. As a result, true medication adherence rates may be lower for the users of corporate and hybrid pharmacies than reported in pharmacy records-based research. Further investigation would need to be conducted to substantiate this hypothesis.

Third, as discussed earlier, the potential lack of generalizability of these findings to other medication classes or health systems is a limitation.

## Conclusion

We found that the type of pharmacy someone uses is a predictor for their likelihood of being adherent to their diabetes medication. The users of corporate and hybrid pharmacies are less likely to be adherent than the users of independent pharmacies, suggesting that prescription copayments, which have been waived by these pharmacies, are not the most significant barrier to medication adherence. Hence, the decision to remove prescription copayments for all New Zealanders may not have a significant effect on the overall rates of medication adherence and access to medicine as intended. Further research into why people choose pharmacies and predictors of adherence not captured by this method will help develop novel strategies to improve rates of medication adherence. ■
